# Treatment of allergic rhinitis with allergen immunotherapy in children and adolescents—Adherence, rhinitis severity, and asthma onset

**DOI:** 10.1111/pai.70304

**Published:** 2026-04-08

**Authors:** Anna M. Hedman, Cecilia Lundholm, Jon R. Konradsen, Åslög Dahl, Maria Ingemansson, Catarina Almqvist

**Affiliations:** ^1^ Department of Medical Epidemiology and Biostatistics Karolinska Institutet Stockholm Sweden; ^2^ Pediatric Allergy and Pulmonology Unit at Astrid Lindgren Children's Hospital, Karolinska University Hospital Stockholm Sweden; ^3^ Department of Biology and Environmental Sciences Gothenburg University Göteborg Sweden

**Keywords:** adherence, allergen immunotherapy, allergic rhinitis, asthma onset, severity

## Abstract

**Background:**

Sublingual immunotherapy (SLIT) has been used to reduce symptoms in allergic rhinitis and to prevent asthma onset. Many studies lack level of adherence and standardized endpoints based on international guidelines.

**Objective:**

We aimed to study the long‐term outcomes of allergic rhinitis severity and asthma onset by adherence of SLIT to grass and birch allergens in children and adolescents with allergic rhinitis.

**Methods:**

In a population‐based register study, we included all children 5–17 years, with initiation of SLIT between 1 July 2006 and 30 June 2022. Allergic rhinitis severity and asthma onset were based on diagnosis and treatment. Adherence was measured for one, two, and 3 years and divided into low, moderate, and high and compared to only one prescription (reference).

**Results:**

7222 children were treated with grass pollen extract and 1184 with birch pollen extract. A significantly lower allergic rhinitis severity was seen in adjusted analyses with odds ratios (OR) of 0.85 (95% confidence interval: 0.72–0.99) and OR 0.81 (0.67–0.96) for the high adherence group (i.e., only one prescription) compared to the reference group in the second and third year respectively after SLIT grass initiation. No statistically significant difference was seen for asthma onset. Estimates for birch SLIT were less conclusive.

**Conclusion:**

We found reduced allergic rhinitis severity with higher adherence and longer treatment duration, particularly for grass SLIT. Shorter follow‐up for birch SLIT and smaller sample size for asthma onset probably influenced our results. The results should encourage clinicians to prescribe SLIT for eligible children and motivate patients to 3‐years full adherence.

**Abbreviations**
AIT: Allergen immunotherapy
CI: Confidence interval
OR: Odds ratio
RCT: Randomized controlled studies
RWD: Real world data
SCIT: Subcutaneous immunotherapy
SLIT: Sublingual immunotherapy

**Key message**

Reduced allergic rhinitis severity was found for grass SLIT with higher adherence and longer treatment duration. This should encourage clinicians to prescribe SLIT for eligible children and motivate patients to full 3‐years adherence.

## 1 BACKGROUND

Allergic rhinitis is an inflammatory disorder triggered by an immune response to inhaled allergens in individuals who are sensitized, with symptoms such as sneezing, itchy or runny nose, and watery eyes.^1^ In a recent meta‐analysis, the prevalence of allergic rhinitis in children ranged between 10% and 18% based on self‐reports and physician‐diagnosis.^2^ Thus, quality of life, social, and economic aspects are all affected for a considerate part of the pediatric population.^2^ The major objective of allergen immunotherapy (AIT) is to achieve immune tolerance to allergens and reduce (or eliminate) symptoms of allergic rhinitis and allergic asthma, with the potential to alter the natural course of allergic disease.^3^ Importantly, AIT has impacted clinical guidelines, regulatory bodies, and care pathways and has been acknowledged by the European Academy of Allergy and Clinical Immunology (EAACI) task force.^4^ AIT with allergens from pollen can be administered subcutaneously (SCIT)^5^ or sublingually (SLIT) as tablets or drops for a treatment period of a minimum of 3 years.^6^ Treatment can begin in individuals from 5 years of age.^7^

Allergic rhinitis is a recognized risk factor for asthma development^8^ and AIT in children has been shown to reduce the risk for developing asthma.^9^, ^10^, ^11^ Randomized controlled studies (RCT) for AIT with SLIT have provided evidence for reduced allergy symptoms in children from the age of 5.^12^, ^13^ Real‐world data‐based studies (RWD) have shown that the benefits of AIT can extend for a long time and in different groups of patients.^14^, ^15^, ^16^ The EAACI task force has recommended amount of medication as one of the primary outcomes in AIT studies,^4^ as well as symptoms and lab‐values.^17^ However, heterogeneity and lack of standardization remain.

Adherence and persistence rates for SLIT have shown a significant variability depending on the methodology and length of follow‐up.^18^, ^19^ RWD studies have the advantage to show the effectiveness of AIT by rates of adherence to therapy, which is difficult to capture in a RCT.^20^ It is yet unknown how adherence to SLIT may impact treatment outcomes such as allergic rhinitis severity and asthma onset.

Previous RWD studies of SLIT have used heterogeneous measures in terms of study population, outcomes, duration of treatment (from one prescription to 3 years) and duration of follow‐up, ranging from 2 to 9 years.^11^, ^21^, ^22^, ^23^ If we are able to show a similar long‐term effect of SLIT in a nationwide register‐based study as previous RCTs, this indicates that continued support for SLIT persistence should be encouraged in the healthcare sector. In this RWD study we know the number of tablets dispensed to each participant and the corresponding treatment duration, enabling classification of adherence into groups based on both the quantity and duration of tablet intake.

### 1.1 Aim

The general aim was to study the long‐term outcomes of allergic rhinitis severity and asthma onset by adherence and treatment duration of sublingual immunotherapy to grass and birch allergens in children and adolescents with allergic rhinitis.

## 2 MATERIALS AND METHODS

### 2.1 Study design and study population

This was a population‐based register study with prospectively recorded data. We included all children between 5 and 17 years who had a dispensed SLIT to grass (Grazax®) and birch pollen (Itulazax®) according to the Swedish prescribed drug registrer (SPDR). The treatment of SLIT should have been initiated between 1st of July 2006 and 30th of June 2022. In Sweden, grass SLIT (Grazax®) has been available for children >5 years since 2009. The birch SLIT (Itulazax®) has been available for adults since 2020^12^ and has recently (May 2025) also been available for children from the age of 5.^13^

### 2.2 Data sources

The SPDR holds data on all prescribed and dispensed medication since 1st of July 2005. Record linkage was possible through the use of the personal identity number, which is a unique identifier assigned to all residents in Sweden. This subsequently allowed linkage across several nationwide registers, including the Swedish Register of the Total Population (RTP). In addition, it allowed further linkage with the National Patient Register (NPR), which includes information on primary and secondary diagnoses according to the International Classification of Diseases (ICD‐10) system from inpatient (since 1987) and outpatient specialist/secondary care (since 2001). Furthermore, the Cause of Death register, which contains information on the date and cause (ICD‐code) of death, the Multi‐generation Register, which allows researchers to link children to their parents, and the Migration register, with information on dates of immigration and emigration, were utilized. Finally, the Longitudinal Integration Database for Health Insurance and Labour Market Studies (LISA), which contains data on education, disposable income, and unemployment, was also applied for linkage. The requirement for obtaining informed consent or parental permission was waived by using the national registers, and data were pseudonymised before delivery to the investigators. The study was approved by the Swedish Ethical Review Authority.

### 2.3 Exposure

SLIT adherence for grass (grass pollen extract) and birch (birch pollen extract) allergens (ATC: V01AA02 and V01AA05 respectively) was measured for the first, second, and third year since treatment initiation of SLIT. Since one SLIT tablet is supposed to be taken each day, we defined adherence as the total amount of tablets dispensed for each individual within the time‐windows of one, two, or 3 years of SLIT treatment divided by the number of days in the time‐window. Four different adherence groups were subsequently created for each time‐window based on the calculated adherence (Table S1): (1) reference (only one dispensed package of SLIT) (2) low adherence (3) moderate adherence and (4) high adherence. In addition, “all” adherence were defined as >1 package.

### 2.4 Outcomes

We used two different outcomes: allergic rhinitis severity and asthma onset.

Allergic rhinitis *severity* was based on the European Forum for Research and Education in Allergy and Airway Diseases (EUFOREA) treatment algorithm.^24^ We defined the following treatment steps based on medications from the SPDR: Treatment step 0; no rhinitis medication. Treatment step 1; nasal corticosteroids (ATC: R01AD05, ‐08, ‐09, ‐11, ‐12) or non‐sedating oral anti‐histamines (ATC: R06AX). Treatment step 2; fixed nasal corticosteroids plus nasal anti‐histamine (ATC: R01AD58, ‐59) or 2 of the following 3: nasal corticosteroids, non‐sedating oral anti‐histamines and ocular anti‐histamines (ATC: S10GX). Treatment step 3; in addition to step 2, any of the following: leukotriene receptor antagonist (ATC: R03DC03), ocular anti‐histamine or short course oral corticosteroids (OCS) (ATC: H02AB01, ‐02, ‐04). Allergic rhinitis severity was measured the year after each time‐window.

*Asthma onset* was investigated in those that did not have any dispensed asthma medications or asthma diagnosis before treatment initiation. Asthma was defined using a validated algorithm which is based on prescribed asthma medications from the SPDR within a certain time window and/or an asthma diagnosis from the NPR (see Appendix S1).^25^ Asthma onset could be present any time after each time window until the end of follow‐up which was 30th of June 2023.

### 2.5 Confounders

Based on previous literature in the field and using directed acyclic graphs (Figure S1a,b) we included the following covariates in our analyses: age in years at the time of SLIT initiation, sex (male/female) of the child and parental country of birth (classified into both parents born in Sweden, one parent born abroad, both parents born abroad) taken from the RTP; socioeconomic status (SES) based on the highest education among the parents (compulsory school, upper secondary school and higher education) derived from LISA; the year of treatment start, parental rhinitis^26^ and parental asthma^25^ derived from both SPDR and NPR based on the same validated algorithms as for the children.

### 2.6 Statistical methods

Background characteristics were estimated with count (%) and mean (SD) for categorical and continuous variables respectively for all individuals and divided into grass and birch allergen. In addition, we separated our cohort into the reference group vs. the adherence groups regarding background characteristics. We applied ordinal logistic regression analysis to estimate the association between adherence to SLIT during the time‐windows of one, two or 3 years and allergic rhinitis severity. We used Cox proportional hazard regression to estimate the association between adherence to SLIT during the time‐window of one, two or 3 years and asthma onset censoring for death, migration, or end of study period (i.e., 30th of June 2023), whichever came first. The analysis timescale was time since treatment initiation with SLIT. We fitted unadjusted models and models adjusted for covariates. In the analyses for asthma onset we adjusted for age, sex, parental country of birth, SES, year of treatment start, parental asthma and parental allergic rhinitis. The standard errors were estimated using the cluster robust sandwich estimator to account for dependency between siblings within the cohort. In the analyses for allergic rhinitis severity we applied the same covariates as for asthma onset, except parental asthma.

For each outcome (i.e., allergic rhinitis severity and asthma onset) we separately analyzed grass‐ and birch allergen extracts. In a combined analysis, we analyzed the first SLIT (grass or birch) called ‘any SLIT’ for each individual. If the number of individuals or events in an exposure group was below five no analysis was performed.

### 2.7 Sensitivity analyses

First, we extracted those individuals who had EUFOREA step nr 3 the year before SLIT initiation and ran the same analyses as above. The rationale was to address severe allergic rhinitis. Secondly, we excluded all individuals that had SLIT treatment for more than one allergen to rule out any treatment interaction.

## 3 RESULTS

In total, 7739 children initiated treatment with SLIT before July 1st 2022. Of these, 529 children were treated for both allergens, respectively. The average age was 12.8 (SD 3.3) years and there were approximately twice as many boys (67%) compared to girls (33%). In regard to each allergen, 88% were treated with grass pollen extract and 12% with birch pollen extract. Parental allergic rhinitis was common, with 52% of all mothers having allergic rhinitis and 38% of all fathers (Table 1). The average age was slightly higher for the treatment initiation of birch SLIT, with a mean of 14.1 (SD 2.8) years, than for the first treatment of grass SLIT, with 12.7 (SD 3.3) years. Allergic rhinitis severity according to EUFOREA treatment step 3 the year before SLIT initiation was 51.7% for grass and 63.5% for birch. In addition, 7222 children in total were treated with grass pollen extract and 1184 with birch pollen extract, with comparable distribution of the baseline characteristics (Table 2). Baseline characteristics when separating the cohort into the reference group vs. the adherence groups is displayed in Table S2.

**TABLE 1:** Baseline characteristics of the cohort by the first allergen.

Baseline, first allergen	All	Grass	Birch
*n* (%)	7739 (100)	6825 (88)	914 (12)
Sex m/f, *n* (%)	5217/2522 (67/33)	4672/2153 (68/32)	545/369 (60/40)
Age, m (SD)	12.8 (3.3)	12.7 (3.3)	14.1 (2.8)
Year of SLIT start, *n* (%)			
2007	21 (0.3)	21 (0.3)	0
2008	57 (0.7)	57 (0.8)	0
2009	322 (4.2)	322 (4.7)	0
2010	411 (5.3)	411 (6.0)	0
2011	410 (5.3)	410 (6.0)	0
2012	400 (5.2)	400 (5.9)	0
2013	321 (4.2)	321 (4.7)	0
2014	356 (4.6)	356 (5.2)	0
2015	423 (5.5)	423 (6.2)	0
2016	534 (6.9)	534 (7.8)	0
2017	551 (7.1)	551 (8.1)	0
2018	574 (7.4)	474 (8.4)	0
2019	661 (8.5)	651 (9.5)	10 (1.1)
2020	1166 (15.1)	805 (11.8)	361 (39.5)
2021	1272 (16.4)	788 (11.6)	484 (53.0)
2022	260 (3.4)	201 (3.0)	59 (6.5)
Swedish born, *n* (%)			
Born in Sweden with both parents born in Sweden	6265 (81)	5575 (81.7)	690 (75.5)
Born in Sweden but one parent born abroad	977 (12.6)	835 (12.2)	142 (15.5)
Born in Sweden with both parents born abroad	497 (6.4)	415 (6.1)	82 (9)
Parental highest education, *n* (%)			
1–9 years	52 (0.7)	44 (0.6)	8 (0.9)
10–12 years	2067 (26.7)	1865 (27.3)	202 (22.1)
>12 years	5612 (72.5)	4908 (71.9)	704 (77)
Missing	8 (0.1)	8 (0.1)	0
Mother allergic rhinitis, *n* (yes/%)	3987/51.5	3507/40.6	480/52.5
Father allergic rhinitis, *n* (yes/%)	2943/38.0	2584/37.9	359/39.3
Mother asthma, *n* (yes/%)	1393/18.0	1186/17.4	207/22.6
Father asthma, *n* (yes/%)	2137/27.6	1856/27.2	281/30.7
Rhinitis severity^a^, EUFOREA, *n* (%)			
Step 0	876 (11.3)	817 (12.0)	59 (6.5)
Step 1	1307 (16.9)	1185 (17.4)	122 (13.4)
Step 2	1451 (18.7)	1298 (19.0)	153 (16.7)
Step 3	4105 (53.0)	3525 (51.7)	580 (63.5)
Asthma^a^, *n* (yes/%)	4105/53.0	3464/50.8	641/70.1

^a^ Year before SLIT initiation.

**TABLE 2:** Baseline characteristics of the cohort for all allergens.

All	Grass	Birch
*n* (%)	7222 (100)	1184 (100)
Sex m/f, *n* (%)	4922/2300 (68/32)	709/475 (60/40)
Age, m (SD)	12.8 (3.3)	14.2 (2.8)
Year of SLIT start, *n* (%)		
2007	21 (0.3)	
2008	57 (0.8)	
2009	326 (4.5)	
2010	418 (5.8)	
2011	416 (5.8)	
2012	404 (5.6)	
2013	328 (4.5)	
2014	356 (4.9)	
2015	427 (5.9)	
2016	537 (7.4)	
2017	559 (7.7)	
2018	588 (8.1)	
2019	662 (9.2)	12 (1)
2020	905 (12.5)	481 (40.6)
2021	951 (13.2)	613 (51.8)
2022	267 (3.7)	78 (6.6)
Swedish born, *n* (%)		
Born in Sweden with both parents born in Sweden	5883 (81.5)	901 (76)
Born in Sweden but one parent born abroad	889 (12.3)	177 (15)
Born in Sweden with both parents born abroad	450 (6.2)	106 (9)
Parental highest education, *n* (%)		
1–9 years	48 (0.7)	9 (0.8)
10–12 years	1952 (27)	257 (21.7)
>12 years	5214 (72.2)	918 (77.5)
Missing	8 (0.1)	0
Mother allergic rhinitis, *n* (yes/%)	3727/51.6	638/53.9
Father allergic rhinitis, *n* (yes/%)	2754/38.1	499/42.1
Mother asthma, *n* (yes/%)	1280/17.7	272/23.0
Father asthma, *n* (yes/%)	1985/27.5	376/31.8
Rhinitis severity^a^, EUFOREA, *n* (%)		
Step 0	846 (11.7)	65 (5.5)
Step 1	1243 (17.2)	176 (14.9)
Step 2	1373 (19.0)	202 (17.1)
Step 3	3760 (52.1)	741 (62.6)
Asthma^a^, *n* (yes/%)	3709/51.4	823/69.5

^a^ Year before SLIT initiation.

### 3.1 Allergic rhinitis severity

#### 3.1.1 Grass pollen SLIT

**FIGURE 1:**
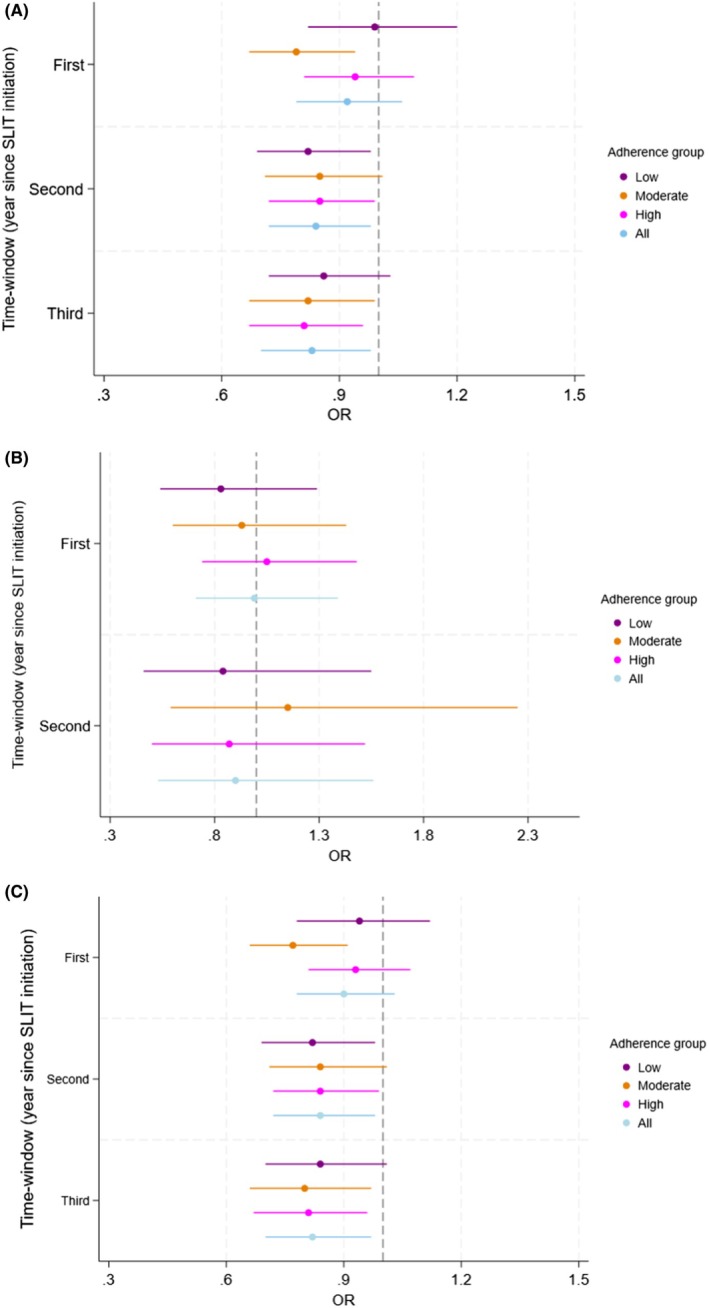
Forest plot; Year of time‐window since treatment initiation of SLIT and allergic rhinitis severity for (A) grass, (B) birch, and (C) Any SLIT (grass or birch) with Odds ratios (OR) and 95% confidence intervals in different adherence groups compared to the reference group adjusted for Age, Sex, Year of treatment start, Socioeconomic status, Parental country of birth and Parental rhinitis; All = Low + Moderate + High.

All estimates were below zero for the adjusted analyses, which indicated a lower severity of allergic rhinitis for all adherence groups compared with those that only had one dispensation (reference), although some estimates were non‐significant, Figure 1A (Table S3a). All adherences had an adjusted OR of 0.92 (0.79–1.06), 0.84 (0.72–0.98), and 0.83 (0.70–0.98) for treatment duration of one, two, and 3 years, respectively.

#### 3.1.2 Birch pollen SLIT

For birch SLIT, the OR estimates were similar to those for grass, but there were no significant results in either time‐window or adherence groups, Figure 1B (Table S3b). An adjusted OR for all adherence of 0.99 (0.71–1.39) and 0.90 (0.53–1.56) for the treatment duration of one and two years, respectively, could be seen.

#### 3.1.3 Any SLIT

When analyzing any SLIT (grass or birch), similar results were found as for grass SLIT in the adjusted analyses, Figure 1C (Table S3c).

### 3.2 Asthma onset

#### 3.2.1 Grass pollen SLIT

**FIGURE 2:**
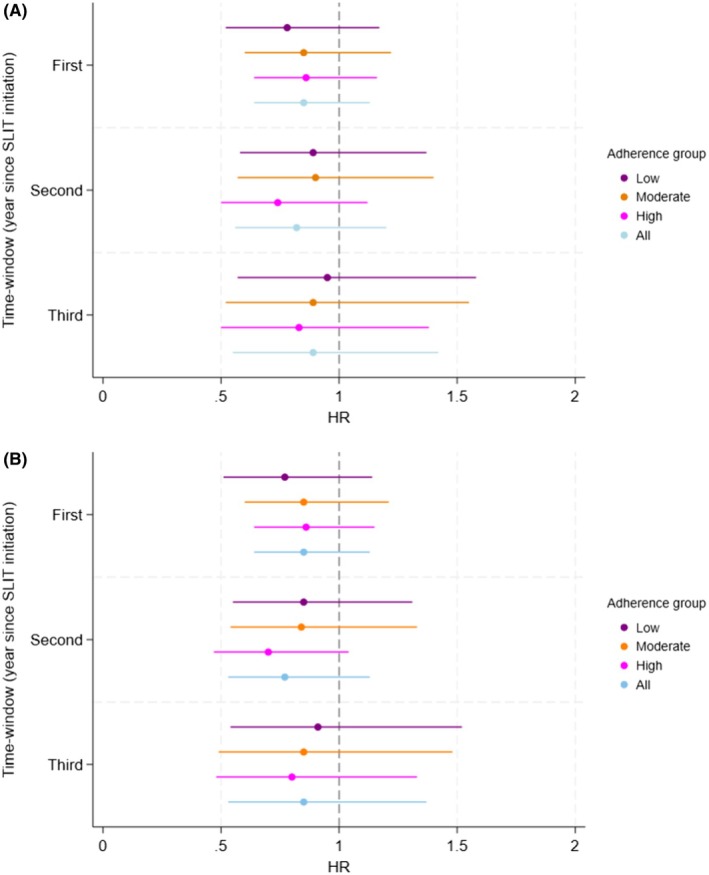
Forest plot; Year of time‐window since treatment initiation of SLIT and asthma onset for (A) grass and (B) Any SLIT (grass or birch) with Hazard ratios (HR) and 95% confidence intervals in different adherence groups compared to the reference group adjusted for Age, Sex, Year of treatment start, Socioeconomic status, Parental country of birth and Parental asthma and rhinitis; All = Low + Moderate + High.

The estimates were all below one for the asthma onset in the different time‐windows and adherence groups compared with the reference group, reflecting a reduction in asthma onset, albeit non‐significant, Figure 2A (Table S4a).

#### 3.2.2 Birch pollen SLIT

No analyses were performed due to the small number of events.

#### 3.2.3 Any SLIT

Similar results as for asthma onset regarding grass SLIT were found for any SLIT (grass or birch), Figure 2B (Table S4b).

### 3.3 Sensitivity analyses

#### 3.3.1 EUFOREA step 3 the year before treatment initiation with SLIT

##### Allergic rhinitis severity

Estimates were similar compared to the main analyses but with fewer significant findings (Table S5a–c). The adjusted analyses for all adherence, grass SLIT, had an OR of 0.85 (0.69–1.05), 0.85 (0.69–1.06), and 0.80 (0.62–1.03) for treatment durations of one, two, and 3 years respectively.

##### Asthma onset

Results were similar compared to the main analyses with a few exceptions (Table S6a,b). The adjusted analyses for all adherence, grass SLIT, had an HR of 0.78 (0.51–1.19) and 0.91 (0.48–1.74) for the treatment durations of one and 2 years, respectively.

#### 3.3.2 Exclusion of individuals treated with more than one allergen

##### Allergic rhinitis severity

Treatment with grass‐, birch‐, and any SLIT (grass or birch) showed similar results as the main analyses with only a few exceptions (Table S7a–c). The adjusted analyses for all adherence, grass SLIT, had an OR of 0.87 (0.75–1.02), 0.81 (0.69–0.95), and 0.82 (0.70–0.97) for treatment durations of one, two, and 3 years respectively.

##### Asthma onset

Results were similar compared to the main analyses (Table S8a,b). The adjusted analyses for all adherence, grass SLIT, had an OR of 0.83 (0.62–1.12), 0.78 (0.53–1.15) and 0.84 (0.52–1.35) for treatment durations of one, two and 3 years respectively.

## 4 DISCUSSION

In this study on adherence and the long‐term consequences of sublingual immunotherapy in children and adolescents with allergic rhinitis, we found a lower allergic rhinitis severity for grass pollen SLIT in the second and third year in the high adherence group compared to the reference group (only one prescription). Similar results were found when we investigated the first allergen of grass or birch.

Several RCTs for both grass^17^, ^27^ and birch^12^, ^28^ SLIT have outcomes in line with our results of lower allergic rhinitis severity, although we could not show significant estimates in the birch SLIT only sample which could be due to a low number of participants mirroring the late introduction in the market for birch pollen extract (Itulazax®) in Sweden. SLIT for grass (Grazax®) has been available for children in Sweden since 2009, reflecting our largest sample of these allergens. In addition, several RWD studies have confirmed and extend the results from RCTs for grass and birch SLIT.^11^, ^21^ RWD plays an important role by complementing RCTs in the outcomes of safety and effectiveness and thereby provides a more comprehensive picture of the ‘real world’, calling for appraisal of the importance of the quality in RWD studies.^29^, ^30^ Here, we expand on the previous findings with a standardized endpoint, and were able to show a lower severity based on allergic rhinitis medications which followed the EUFOREA treatment algorithm.^24^

Regarding asthma onset for those that did not have asthma nor any asthma medication before SLIT initiation, we were not able to corroborate previous findings of reduced asthma onset after initiation of immunotherapy.^17^, ^23^, ^31^, ^32^ However, our point estimates were all below zero for the adjusted analyses in the grass SLIT cohort indicating a reduction, but confidence intervals included one and sample size were smaller compared to the analyses of allergic rhinitis severity. Therefore we can not draw any conclusions regarding asthma onset.

A prerequisite for successful AIT is adherence and therefore a key challenge to ensure effectiveness.^11^ International guidelines regarding AIT recommend a minimum of 3 years of treatment^33^ and RCTs have demonstrated that 3 years of continuous SLIT or SCIT have resulted in long‐term clinical outcomes such as reduced symptoms and reduced antiallergic medications after at least 2 years of discontinuation.^34^, ^35^ As expected, we found decreased adherence with each year of treatment duration for all allergens compared with the reference group (i.e., only one prescription). We found adherence in relation to long‐term outcome, in particular for our largest cohort of grass SLIT, a decrease in allergic rhinitis severity the second and third year of treatment in the highest adherence group compared to the reference group. This is in line with reports which state that it generally takes at least 1 year for full treatment effects to become evident. Studies that assessed the 3 years' time window for AIT reported that AIT adherence decreased.^11^, ^36^, ^37^, ^38^ Furthermore, clinical trials have reported higher adherence for AIT than real‐world data^6^ but conditions vary completely between the two kinds of studies. Thus, RCTs usually follow a strict protocol and participants are often surveilled and perhaps even paid to partake, all which influence adherence.^6^ Several factors influence medication adherence in AIT such as inconvenience, improvement without treatment and perception of poor efficacy.^39^ Other formulas of SLIT are currently being introduced and need to be evaluated in the future.

Strengths of our study include nation‐wide register‐based data with prospectively recorded objective data in a standardized way with possibility to evaluate outcomes after several years. Furthermore, several registers were linked to each other and we were therefore able to include important covariates to adjust for in our analyses to reduce confounding. Importantly, another strength was inclusion of different adherence groups (i.e., low, moderate, high) in terms of complete/partial coverage of dispensed medication for various time‐periods. Regarding limitations; we did not use controls that were never treated with SLIT, but instead used those that only had one dispensed package (reference group) and we do not have information on whether a child experienced symptom reduction and therefore stopped treatment or whether it was discontinued for other reasons. Studies have however shown that symptom reduction can occur within the first 3–6 months, with full maximal clinical effect after 1 year.^33^ Although we had a relatively large population in comparision with other studies we were not able to show a statistically significantly lower asthma onset and birch pollen‐ SLIT was fairly recently introduced to the Swedish market which also influenced the total number of participants and hence the statistical power. Moreover, we did not use any objective markers such as lab‐values or skin‐prick test and did not have data on over‐the‐counter medication but instead used a validated algorithm for the outcome asthma onset^25^ and severity measures of allergic rhinitis based on international guidelines (EUFOREA) and dispensed medication recorded in the SPDR. Finally, we do not know the real sequence of symptom occurrence and the choice of treatment. Being categorized into the highest allergic rhinitis severity step (EUFOREA 3) may be due to severe allergy or just being prone to dispensing the prescribed medications.

Our results are highly relevant to the clinics and society. A review reported that over a longer time period, AIT is more cost‐effective than pharmacotherapy.^40^ The cost savings with AIT appear to be due to decreased pharmacotherapy use in terms of allergic rhinitis medication and decreased asthma‐related healthcare. In this light, our results could add important knowledge to future decisions made by policy makers.

## 5 CONCLUSION

We found a clear result of lower allergic rhinitis severity, particularly for grass SLIT and especially for higher adherence and longer follow‐up. This should encourage clinicians to prescribe SLIT for children and motivate patients for 3‐years full adherence for improvements. Furthermore, health care personnel should be educated of the treatment benefits and that adherence is of importance for the best long‐term outcome.

## AUTHOR CONTRIBUTIONS

**Catarina Almqvist:** Conceptualization; investigation; funding acquisition; methodology; visualization; writing – review and editing; project administration; supervision; resources; validation. **Cecilia Lundholm:** Supervision; data curation; software; writing – review and editing; visualization; validation; methodology; conceptualization; investigation. **Åslög Dahl:** Writing – review and editing; validation; project administration; resources; investigation; conceptualization. **Maria Ingemansson:** Investigation; writing – review and editing; project administration; resources; validation; conceptualization. **Jon R. Konradsen:** Conceptualization; methodology; visualization; writing – review and editing; investigation; resources. **Anna M. Hedman:** Conceptualization; investigation; writing – original draft; methodology; validation; visualization; writing – review and editing; project administration; data curation; formal analysis; software; resources.

## FUNDING INFORMATION

Funding was provided from the Swedish Research Council (grant no 2023–02327), the Swedish Heart‐Lung Foundation (grant no 20240974), the Swedish Asthma and Allergy Association Research Fund (grant no 2024–0010), grants provided by Region Stockholm (ALF project RS2022‐0674), the Strategic Research Program in Epidemiology at Karolinska Institutet, and the Foundation Frimurare Barnhuset in Stockholm.

## CONFLICT OF INTEREST STATEMENT

CA has been a steering group member for the Swedish Pediatric Association, section for allergology and respiratory medicine. JK has been on the advisory boards for Novartis and ALK. MI has given lectures to ALK.

## Supporting information


Figure S1.

Appendix S1.

Table S1.
