# Supplementary material for: Treatment of allergic rhinitis with allergen immunotherapy in children and adolescents—Adherence, rhinitis severity, and asthma onset
Source: Pediatr Allergy Immunol. 2026 Apr 8;37(4):e70304. doi: 10.1111/pai.70304 (PMC13062723; doi:10.1111/pai.70304)
Supplement: Supplementary file 1 — Figure S1. [file PAI-37-e70304-s003.docx]

**Online Repository Figures**

**DAGs**

**
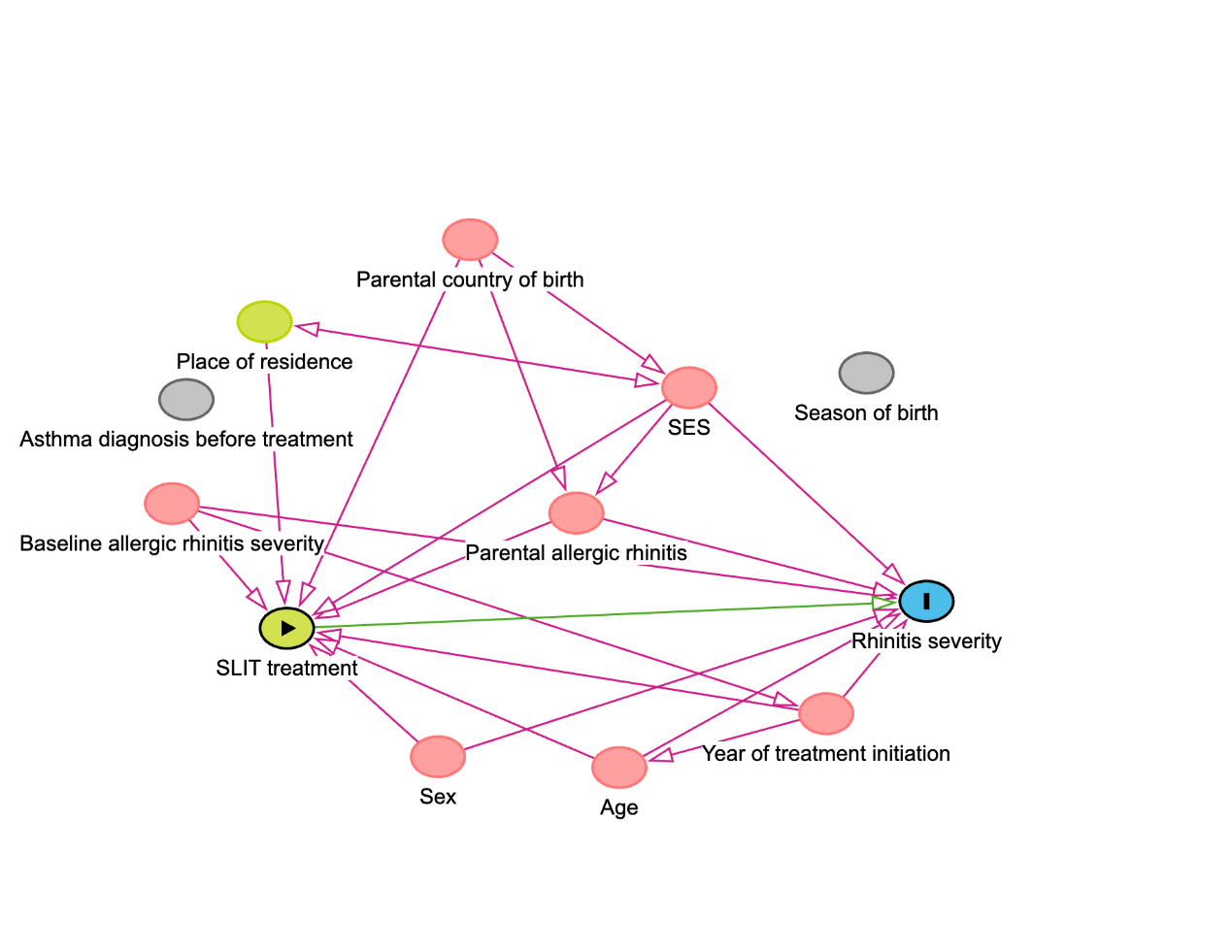
**

**Figure S1a) DAG: Association between adherence of SLIT treatment and allergic rhinitis severity.** SES=Socioeconomic status


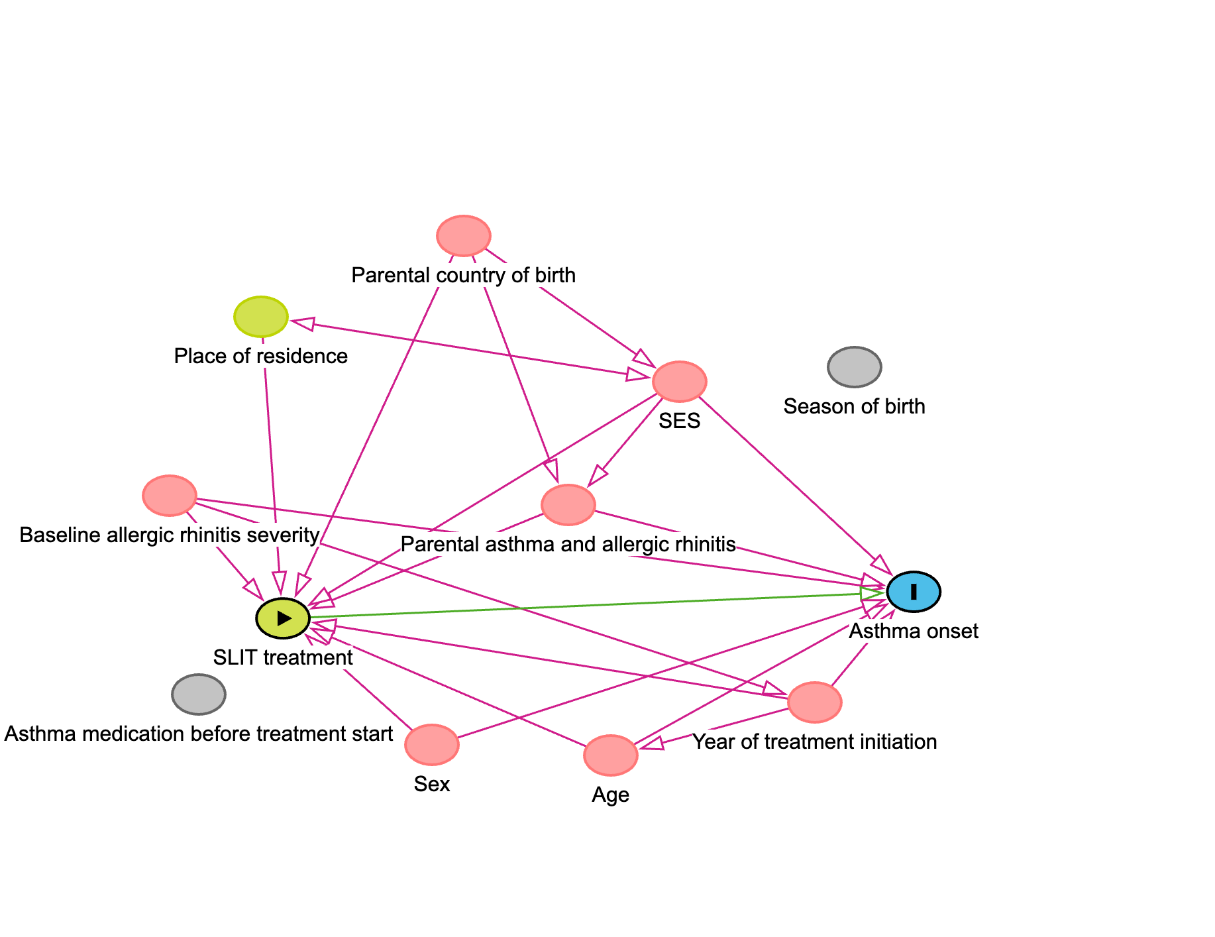


**Figure S1b) DAG: Association between adherence of SLIT treatment and asthma onset.** SES=Socioeconomic status
