# Supplementary material for: Treatment of allergic rhinitis with allergen immunotherapy in children and adolescents—Adherence, rhinitis severity, and asthma onset
Source: Pediatr Allergy Immunol. 2026 Apr 8;37(4):e70304. doi: 10.1111/pai.70304 (PMC13062723; doi:10.1111/pai.70304)
Supplement: Supplementary file 2 — Appendix S1. [file PAI-37-e70304-s002.docx]

**Online Repository**

**Appendix S1**

Anna M Hedman PhD^1^, Cecilia Lundholm PhD^1^, Jon R Konradsen MD, PhD^1,2^, Åslög Dahl PhD^3^, Maria Ingemarsson MD, PhD^2^, Catarina Almqvist MD, PhD^1,2^

1. Department of Medical Epidemiology and Biostatistics, Karolinska Institutet, Stockholm, Sweden
2. Pediatric Allergy and Pulmonology Unit at Astrid Lindgren Children's Hospital, Karolinska University Hospital, Stockholm, Sweden
3. Department of Biology and Environmental Sciences, Gothenburg University

**Corresponding author:**
Anna M Hedman
Dept of Medical Epidemiology and Biostatistics
Karolinska Institutet

Nobels väg 12A
171 77 Stockholm

Sweden

[Anna.Hedman@ki.se](mailto:Anna.Hedman@ki.se)

Asthma algorithm

According to Örtqvist et al (1) for children and adolecsents >4.5 to 17 years: an asthma diagnosis (ICD 10: J45) and/or ≥ 2 of ICS (ATC: R03BA) and/or LRTA (ATC: R03DC) and/or β2-ICS (ATC: R03AK), independent of time between distributions and/or ≥ 3 of ICS (ATC: R03BA) and/or LRTA (ATC: R03DC) and/or β2-ICS (ATC: R03AK) and/or β2 (ATC: R03AC), within a 12-month-period.

1. Ortqvist AK, Lundholm C, Wettermark B, Ludvigsson JF, Ye W, Almqvist C. Validation of asthma and eczema in population-based Swedish drug and patient registers. Pharmacoepidemiol Drug Saf. 2013;22(8):850-60.
