# Supplementary material for: Treatment of allergic rhinitis with allergen immunotherapy in children and adolescents—Adherence, rhinitis severity, and asthma onset
Source: Pediatr Allergy Immunol. 2026 Apr 8;37(4):e70304. doi: 10.1111/pai.70304 (PMC13062723; doi:10.1111/pai.70304)
Supplement: Supplementary file 3 — Table S1. [file PAI-37-e70304-s001.docx]

**Online Repository Tables**

**Table S1. Adherence groups** based on the total amount of dispensed SLIT tablets for each allergen and time-window.

| **Adherence-group** | **Time-window** | | |
| --- | --- | --- | --- |
|  | **one year** | **two years** | **three years** |
| **Grass** | *total amount of tablets* | | |
| *Reference* | 30 or 100 | 30 or 100 | 30 or 100 |
| *Low* | 30<to<230 | 30<to<430 | 30<to<630 |
| *Moderate* | 230<=to<330 | 430<=to<630 | 630<=to<900 |
| *High* | >=330 | >=630 | >=900 |
| **Birch** | *total amount of tablets* | | |
| *Reference* | 30 or 90 | 30 or 90 | 30 or 90 |
| *Low* | 30<to<210 | 30<to<390 | 30<to<570 |
| *Moderate* | 210<=to<300 | 390<=to<570 | 570<=to<870 |
| *High* | >=300 | >=570 | >=870 |
|  |  | | |
| Grass (Grazax) is dispensed in a package with 30 (start packages) or 100 tables | | | |
| Birch (Itulazax) is dispensed in a package with 30 (start packages) or 90 tablets | | | |
|  | | |  |
|  | |  |  |

Within each time-window: Low adherence was based on more than one dispensed package. Moderate adherence was based on more than two dispensed packages. High adherence was based on more than three dispensed packages.

**Table S2. Baseline characteristics divided into the reference and all adherence, grass and birch**

|  | **Grass** | | **Birch** | |
| --- | --- | --- | --- | --- |
|  | Ref One dispensation | All adherence | Ref One dispensation | All adherence |
| **n (%)** | 848 (11.7) | 6374 (88.3) | 148 (12.5) | 1036 (87.5) |
| **Sex male, n(%)** | 563 (66) | 4359 (68) | 92 (62) | 617 (60) |
| **Age, m (SD)** | 13.7 (3.1) | 12.6 (3.3) | 14.5 (2.4) | 14.1 (2.8) |
| **Year of SLIT start, n (%)** |  |  |  |  |
| **2007** | 1 (0.1) | 20 (0.3) |  |  |
| **2008** | 10 (1.2) | 47 (0.7) |  |  |
| **2009** | 36 (4.3) | 290 (4.6) |  |  |
| **2010** | 57 (6.7) | 361 (5.7) |  |  |
| **2011** | 55 (6.5) | 361 (5.7) |  |  |
| **2012** | 52 (6.1) | 352 (5.5) |  |  |
| **2013** | 45 (5.3) | 283 (4.4) |  |  |
| **2014** | 42 (5.0) | 314 (4.9) |  |  |
| **2015** | 48 (5.7) | 379 (6.0) |  |  |
| **2016** | 61 (7.2) | 476 (7.5) |  |  |
| **2017** | 85 (10.0) | 474 (7.4) |  |  |
| **2018** | 75 (8.8) | 513 (8.1) |  |  |
| **2019** | 87 (10.3) | 575 (9.0) | 2 (1.4) | 10 (1.0) |
| **2020** | 76 (9.0) | 829 (13.0) | 54 (36.5) | 427 (41.2) |
| **2021** | 90 (10.6) | 861 (13.5) | 84 (56.8) | 529 (51.1) |
| **2022** | 28 (3.3) | 239 (3.8) | 8 (5.4) | 70 (6.8) |
| **Swedish born, n (%)** |  |  |  |  |
| Born in Sweden with both parents born in Sweden | 661 (78.0) | 5222 (81.9) | 99 (66.9) | 802 (77.4) |
| Born in Sweden but one parent born abroad | 98 (11.6) | 791 (12.4) | 32 (21.6) | 145 (14.0) |
| Born in Sweden with both parents born abroad | 89 (10.5) | 361 (5.7) | 17 (11.5) | 89 (8.6) |
|  |  |  |  |  |
| **Parental highest education, n (%)** |  |  |  |  |
| 1-9 years | 11 (1.3) | 37 (0.6) | 4 (2.7) | 5 (0.5) |
| 10-12 years | 303 (35.7) | 1649 (25.9) | 45 (30.4) | 212 (20.5) |
| >12 years | 532 (62.7) | 4682 (73.5) | 99 (66.9) | 819 (79.1) |
| Missing | 2 (0.2) | 6 (0.1) | 0 | 0 |
| **Mother allergic rhinitis,** n (yes/%) | 426/50.2 | 3301/51.8 | 90/60.8 | 548/52.9 |
| **Father allergic rhinitis,** n (yes/%) | 319/37.6 | 2435/38.2 | 61/41.2 | 438/42.3 |
| **Mother asthma**, n (yes/%) | 160/18.9 | 1120/17.6 | 39/26.4 | 233/22.5 |
| **Father asthma,** n (yes/%) | 241/28.4 | 1744/27.4 | 48/32.4 | 328/31.7 |
| **Asthma*,** n (yes/%) | 432/50.9 | 3277/51.4 | 110/74.3 | 713/68.9 |
| **EUFOREA** year before SLIT, n (%) |  |  |  |  |
| **0** | 105 (12.4) | 741 (11.6) | 11 (7.4) | 54 (5.2) |
| **1** | 163 (19.2) | 1080 (16.9) | 27 (18.2) | 149 (14.4) |
| **2** | 152 (17.9) | 1221 (19.2) | 29 (19.6) | 173 (16.7) |
| **3** | 428 (50.5) | 3332 (52.3) | 81 (54.7) | 660 (63.7) |
| *Asthma before baseline |  |  |  |  |

**Table S3. Allergic rhinitis severity for different adherence groups**

1. **Table E3a: Grass pollen extract**

|  |  | **EUFOREA** | |  | Unadjusted | | Adjusted* |  |  |
| --- | --- | --- | --- | --- | --- | --- | --- | --- | --- |
| **One year time-window** | **0** | **1** | **2** | **3** | **OR** | **95% (CI)** | **OR** | **95% (CI)** |  |
| **Ref one dispensation** | 281 | 166 | 116 | 285 | **1** |  |  |  |  |
| Low adherence | 267 | 186 | 128 | 285 | 1.04 | 0.87-1.23 | 0.99 | 0.82-1.20 |  |
| Moderate adherence | 406 | 289 | 220 | 337 | 0.88 | 0.75-1.03 | **0.79** | **0.67-0.94** |  |
| High adherence | 1155 | 1011 | 823 | 1260 | 1.06 | 0.93-1.22 | 0.94 | 0.81-1.09 |  |
| All adherence | 1828 | 1486 | 1171 | 1882 | 1.02 | 0.90-1.17 | 0.92 | 0.79-1.06 |  |
|  |  | **EUFOREA** | |  | Unadjusted | | Adjusted* |  |  |
| **Two years time-window** | **0** | **1** | **2** | **3** | **OR** | **95% (CI)** | **OR** | **95% (CI)** |  |
| **Ref one dispensation** | 263 | 130 | 98 | 217 | **1** |  |  |  |  |
| Low adherence | 603 | 319 | 212 | 412 | 0.86 | 0.73-1.02 | **0.82** | **0.69-0.98** |  |
| Moderate adherence | 431 | 296 | 222 | 329 | 0.98 | 0.82-1.15 | 0.85 | 0.71-1.01 |  |
| High adherence | 887 | 631 | 522 | 709 | 1.02 | 0.88-1.19 | **0.85** | **0.72-0.99** |  |
| All adherence | 1921 | 1246 | 956 | 1450 | 0.97 | 0.84-1.12 | **0.84** | **0.72-0.98** |  |
|  |  | **EUFOREA** | |  | Unadjusted | | Adjusted* |  |  |
| **Three years time-window** | **0** | **1** | **2** | **3** | **OR** | **95% (CI)** | **OR** | **95% (CI)** |  |
| **Ref one dispensation** | 267 | 115 | 88 | 143 | **1** |  |  |  |  |
| Low adherence | 806 | 326 | 253 | 377 | 0.91 | 0.77-1.08 | 0.86 | 0.72-1.03 |  |
| Moderate adherence | 454 | 200 | 174 | 236 | 1.01 | 0.84-1.22 | **0.82** | **0.67-0.99** |  |
| High adherence | 773 | 396 | 313 | 390 | 1.01 | 0.86-1.20 | **0.81** | **0.67-0.96** |  |
| All adherence | 2033 | 922 | 740 | 1003 | 0.97 | 0.83-1.14 | **0.83** | **0.70-0.98** |  |
| *Age, Sex, Year of treatment start, SES, Parental country of birth, Parental rhinitis (cluster robost sandwich estimator)  EUFOREA= European Forum for Research and Education in Allergy and Airway Diseases treatment algorithm, OR=Odds Ratio, CI=Confidence Interval, SES=Socioeconomic status   1. **Table E3b: Birch pollen extract**  \| **Birch** \|  \|  \|  \|  \|  \| **ALLERGIC RHINITIS SEVERITY** \| \| \| \| --- \| --- \| --- \| --- \| --- \| --- \| --- \| --- \| --- \| \|  \|  \| **EUFOREA** \| \|  \| Unadjusted \| \| Adjusted* \|  \| \| **One year time-window** \| **0** \| **1** \| **2** \| **3** \| **OR** \| **95% (CI)** \| **OR** \| **95% (CI)** \| \| **Ref one dispensation** \| 39 \| 29 \| 28 \| 52 \| **1** \|  \|  \|  \| \| Low adherence \| 45 \| 38 \| 26 \| 49 \| 0.82 \| 0.54-1.23 \| 0.83 \| 0.54-1.29 \| \| Moderate adherence \| 44 \| 33 \| 31 \| 52 \| 0.91 \| 0.60-1.37 \| 0.93 \| 0.61-1.43 \| \| High adherence \| 150 \| 190 \| 134 \| 243 \| 1.04 \| 0.76-1.44 \| 1.05 \| 0.74-1.48 \| \| All adherence \| 239 \| 261 \| 191 \| 344 \| 0.99 \| 0.72-1.35 \| 0.99 \| 0.71-1.39 \| \|  \|  \| **EUFOREA** \| \|  \| Unadjusted \| \| Adjusted* \|  \| \| **Two years time-window** \| **0** \| **1** \| **2** \| **3** \| **OR** \| **95% (CI)** \| **OR** \| **95% (CI)** \| \| **Ref one dispensation** \| 22 \| 11 \| 8 \| 19 \| **1** \|  \|  \|  \| \| Low adherence \| 59 \| 28 \| 12 \| 43 \| 0.82 \| 0.47-1.43 \| 0.84 \| 0.46-1.55 \| \| Moderate adherence \| 28 \| 21 \| 12 \| 30 \| 1.15 \| 0.63-2.10 \| 1.15 \| 0.59-2.25 \| \| High adherence \| 83 \| 71 \| 41 \| 63 \| 0.93 \| 0.56-1.56 \| 0.87 \| 0.50-1.52 \| \| All adherence \| 170 \| 120 \| 65 \| 136 \| 0.94 \| 0.57-1.53 \| 0.90 \| 0.53-1.56 \|   *Age, Sex, Year of treatment start, SES, Parental country of birth, Parental rhinitis (cluster robost sandwich estimator)  EUFOREA= European Forum for Research and Education in Allergy and Airway Diseases treatment algorithm, OR=Odds Ratio, CI=Confidence Interval, SES=Socioeconomic status. | | | | | | | | | |

1. **Table E3c: Any SLIT (grass or birch)**

| **Any SLIT (grass or birch)** | |  |  |  |  | **ALLERGIC RHINITIS SEVERITY** | | |
| --- | --- | --- | --- | --- | --- | --- | --- | --- |
|  | **EUFOREA** | | | | Unadjusted | | Adjusted* | |
| **One year time-window** | **0** | **1** | **2** | **3** | **OR** | **95% (CI)** | **OR** | **95% (CI)** |
| **Ref one dispensation** | 292 | 182 | 140 | 312 | **1** |  |  |  |
| Low adherence | 294 | 199 | 146 | 304 | 0.97 | 0.82-1.14 | 0.94 | 0.78-1.12 |
| Moderate adherence | 422 | 303 | 226 | 351 | **0.84** | **0.72-0.94** | **0.77** | **0.66-0.91** |
| High adherence | 1224 | 1087 | 878 | 1372 | 1.03 | 0.90-1.17 | 0.93 | 0.81-1.07 |
| All adherence | 1940 | 1589 | 1250 | 2027 | 0.98 | 0.86-1.11 | 0.90 | 0.78-1.03 |
|  | EUFOREA | | | | Unadjusted | | Adjusted* | |
| **Two years time-window** | 0 | 1 | 2 | 3 | **OR** | **95% (CI)** | **OR** | **95% (CI)** |
| **Ref one dispensation** | 271 | 134 | 99 | 228 | **1** |  |  |  |
| Low adherence | 627 | 321 | 215 | 429 | **0.85** | **0.72-0.99** | **0.82** | **0.69-0.98** |
| Moderate adherence | 440 | 307 | 222 | 335 | 0.96 | 0.81-1.13 | 0.84 | 0.71-1.01 |
| High adherence | 922 | 665 | 536 | 733 | 1.00 | 0.86-1.17 | **0.84** | **0.72-0.99** |
| All adherence | 1989 | 1293 | 973 | 1497 | 0.95 | 0.82-1.10 | **0.84** | **0.72-0.98** |
|  | EUFOREA | | | | Unadjusted | | Adjusted* | |
| **Three years time-window** | 0 | 1 | 2 | 3 | **OR** | **95% (CI)** | **OR** | **95% (CI)** |
| **Ref one dispensation** | 259 | 117 | 75 | 152 | **1** |  |  |  |
| Low adherence | 804 | 327 | 228 | 399 | 0.89 | 0.75-1.06 | 0.84 | 0.70-1.01 |
| Moderate adherence | 460 | 203 | 157 | 254 | 0.99 | 0.82-1.19 | **0.80** | **0.66-0.97** |
| High adherence | 766 | 394 | 277 | 429 | 1.01 | 0.86-1.20 | **0.81** | **0.67-0.96** |
| All adherence | 2030 | 924 | 662 | 1082 | 0.96 | 0.82-1.12 | **0.82** | **0.70-0.97** |
| *Age, Sex, Year of treatment start, SES, Parental country of birth, Parental rhinitis (cluster robost sandwich estimator)  EUFOREA= European Forum for Research and Education in Allergy and Airway Diseases treatment algorithm, OR=Odds Ratio, CI=Confidence Interval, SES=Socioeconomic status  **Table S4. Asthma onset for different adherence groups**   1. **Table E4a: Grass pollen extract**  \| **Grass** \|  \|  \|  \| **ASTHMA ONSET** \| \|  \| \| --- \| --- \| --- \| --- \| --- \| --- \| --- \| \|  \|  \|  \| Unadjusted \| \| Adjusted* \| \| \| **One year time-window** \| **No Asthma** \| **Asthma** \| **HR** \| **95% (CI)** \| **HR** \| **95% (CI)** \| \| **Ref one dispensation** \| 358 \| 53 \| **1** \|  \|  \|  \| \| Low adherence \| 328 \| 34 \| 0.80 \| 0.54-1.19 \| 0.78 \| 0.52-1.17 \| \| Moderate adherence \| 504 \| 70 \| 0.96 \| 0.68-1.36 \| 0.85 \| 0.60-1.22 \| \| High adherence \| 1854 \| 265 \| 1.00 \| 0.75-1.32 \| 0.86 \| 0.64-1.16 \| \| All adherence \| 2686 \| 369 \| 0.97 \| 0.73-1.27 \| 0.85 \| 0.64-1.13 \| \|  \|  \|  \| Unadjusted \| \| Adjusted* \| \| \| **Two years time-window** \| **No Asthma** \| **Asthma** \| **HR** \| **95% (CI)** \| **HR** \| **95% (CI)** \| \| **Ref one dispensation** \| 322 \| 32 \| **1** \|  \|  \|  \| \| Low adherence \| 618 \| 55 \| 0.98 \| 0.64-1.49 \| 0.89 \| 0.58-1.37 \| \| Moderate adherence \| 556 \| 52 \| 1.11 \| 0.72-1.70 \| 0.90 \| 0.57-1.40 \| \| High adherence \| 1308 \| 115 \| 0.95 \| 0.65-1.39 \| 0.74 \| 0.50-1.12 \| \| All adherence \| 2482 \| 222 \| 0.99 \| 0.69-1.42 \| 0.82 \| 0.56-1.20 \| \|  \|  \|  \| Unadjusted \| \| Adjusted* \| \| \| **Three years time-window** \| **No Asthma** \| **Asthma** \| **HR** \| **95% (CI)** \| **HR** \| **95% (CI)** \| \| **Ref one dispensation** \| 293 \| 21 \| **1** \|  \|  \|  \| \| Low adherence \| 732 \| 53 \| 1.07 \| 0.65-1.76 \| 0.95 \| 0.57-1.58 \| \| Moderate adherence \| 494 \| 37 \| 1.13 \| 0.67-1.92 \| 0.89 \| 0.52-1.55 \| \| High adherence \| 932 \| 68 \| 1.08 \| 0.67-1.76 \| 0.83 \| 0.50-1.38 \| \| All adherence \| 2158 \| 158 \| 1.09 \| 0.69-1.71 \| 0.89 \| 0.55-1.42 \| \| *Age, Sex, Year of treatment start, SES, Parental country of birth, Parental asthma and rhinitis and relatedness (cluster robost sandwich estimator). HR=Hazard Ratio, CI=Confidence Interval, SES=Socioeconomic status   \|  \| \| --- \| \| \| \| \| \| \| \| | | | | | | | | |

1. **Table E4b: Any SLIT (grass or birch)**

| **Any SLIT (grass or birch)** |  |  |  | **ASTHMA ONSET** | |  |
| --- | --- | --- | --- | --- | --- | --- |
|  |  |  | Unadjusted | | Adjusted* | |
| **One year time-window** | **No Asthma** | **Asthma** | **HR** | **95% (CI)** | **HR** | **95% (CI)** |
| **Ref one dispensation** | 369 | 54 | **1** |  |  |  |
| Low adherence | 344 | 35 | 0.79 | 0.53-1.17 | 0.77 | 0.51-1.14 |
| Moderate adherence | 515 | 71 | 0.95 | 0.67-1.33 | 0.85 | 0.60-1.21 |
| High adherence | 1931 | 267 | 0.98 | 0.74-1.29 | 0.86 | 0.64-1.15 |
| All adherence | 2790 | 373 | 0.95 | 0.72-1.25 | 0.85 | 0.64-1.13 |
|  |  |  | Unadjusted | | Adjusted* | |
| **Two years time-window** | **No Asthma** | **Asthma** | **HR** | **95% (CI)** | **HR** | **95% (CI)** |
| **Ref one dispensation** | 319 | 33 | **1** |  |  |  |
| Low adherence | 627 | 55 | 0.93 | 0.61-1.41 | 0.85 | 0.55-1.31 |
| Moderate adherence | 561 | 51 | 1.04 | 0.68-1.58 | 0.84 | 0.54-1.33 |
| High adherence | 1333 | 113 | 0.88 | 0.60-1.29 | 0.70 | 0.47-1.04 |
| All adherence | 2521 | 219 | 0.93 | 0.65-1.33 | 0.77 | 0.53-1.13 |
|  |  |  | Unadjusted | | Adjusted* | |
| **Three years time-window** | **No Asthma** | **Asthma** | **HR** | **95% (CI)** | **HR** | **95% (CI)** |
| **Ref one dispensation** | 286 | 21 | **1** |  |  |  |
| Low adherence | 726 | 51 | 1.01 | 0.61-1.67 | 0.91 | 0.54-1.52 |
| Moderate adherence | 495 | 36 | 1.07 | 0.63-1.82 | 0.85 | 0.49-1.48 |
| High adherence | 927 | 67 | 1.04 | 0.64-1.68 | 0.80 | 0.48-1.33 |
| All adherence | 2148 | 154 | 1.04 | 0.66-1.63 | 0.85 | 0.53-1.37 |
| *Age, Sex, Year of treatment start, SES, Parental country of birth, Parental asthma and rhinitis and relatedness (cluster robost sandwich estimator). HR=Hazard Ratio, CI=Confidence Interval, SES=Socioeconomic status | | | | | | |

Sensitivity-analyses

EUFOREA nr 3 before treatment initiation of SLIT

**Table S5. Allergic rhinitis severity for different adherence groups**

1. **Table E4a: Grass**

| SENSITIVITY ANALYSIS: EUFOREA nr 3 year before treatment initiation | | | | | |  |  |  |
| --- | --- | --- | --- | --- | --- | --- | --- | --- |
| **Grass** |  |  |  |  |  | **ALLERGIC RHINITIS SEVERITY** | |  |
|  |  | **EUFOREA** | |  | Unadjusted | | Adjusted* | |
| **One year time-window** | **0** | **1** | **2** | **3** | **OR** | **95% (CI)** | **OR** | **95% (CI)** |
| **Ref one dispensation** | 114 | 57 | 57 | 200 | **1** |  |  |  |
| Low adherence | 119 | 76 | 59 | 205 | 0.94 | 0.73-1.20 | 0.91 | 0.69-1.19 |
| Moderate adherence | 151 | 134 | 114 | 237 | 0.82 | 0.65-1.02 | **0.73** | **0.57-0.94** |
| High adherence | 440 | 448 | 409 | 935 | 1.00 | 0.82-1.21 | 0.88 | 0.71-1.09 |
| All adherence | 710 | 658 | 582 | 1377 | 0.95 | 0.79-1.15 | 0.85 | 0.69-1.05 |
|  |  | **EUFOREA** | |  | Unadjusted | | Adjusted* | |
| **Two years time-window** | **0** | **1** | **2** | **3** | **OR** | **95% (CI)** | **OR** | **95% (CI)** |
| **Ref one dispensation** | 103 | 55 | 53 | 141 | **1** |  |  |  |
| Low adherence | 227 | 146 | 113 | 274 | 0.88 | 0.69-1.10 | 0.84 | 0.66-1.07 |
| Moderate adherence | 166 | 136 | 105 | 240 | 0.98 | 0.77-1.24 | 0.86 | 0.67-1.10 |
| High adherence | 339 | 264 | 267 | 512 | 1.03 | 0.83-1.28 | 0.86 | 0.68-1.08 |
| All adherence | 732 | 546 | 485 | 1026 | 0.96 | 0.79-1.20 | 0.85 | 0.69-1.06 |
|  |  | **EUFOREA** | |  | Unadjusted | | Adjusted* | |
| **Three years time-window** | **0** | **1** | **2** | **3** | **OR** | **95% (CI)** | **OR** | **95% (CI)** |
| **Ref one dispensation** | 110 | 44 | 32 | 107 | **1** |  |  |  |
| Low adherence | 312 | 136 | 135 | 256 | 0.90 | 0.71-1.16 | 0.85 | 0.65-1.11 |
| Moderate adherence | 180 | 83 | 89 | 164 | 0.99 | 0.76-1.29 | 0.78 | 0.59-1.04 |
| High adherence | 282 | 169 | 168 | 258 | 0.99 | 0.78-1.26 | **0.76** | **0.60-0.99** |
| All adherence | 774 | 388 | 392 | 678 | 0.95 | 0.76-1.20 | 0.80 | 0.62-1.03 |
| *Age, Sex, Year of treatment start, SES, Parental country of birth, Parental rhinitis (cluster robost sandwich estimator) | | | | | | | | |

EUFOREA= European Forum for Research and Education in Allergy and Airway Diseases treatment algorithm, OR=Hazard Ratio, CI=Confidence Interval, SES=Socioeconomic status

1. **Table E5b: Birch**

| SENSITIVITY ANALYSIS: EUFOREA nr 3 year before treatment initiation | | | | | |  |  |  |  |
| --- | --- | --- | --- | --- | --- | --- | --- | --- | --- |
| **Birch** |  |  |  |  |  | **ALLERGIC RHINITIS SEVERITY** | |  |  |
|  |  | **EUFOREA** | |  | Unadjusted | | Adjusted* | |  |
| **One year time-window** | **0** | **1** | **2** | **3** | **OR** | **95% (CI)** | **OR** | **95% (CI)** |  |
| **Ref one dispensation** | 15 | 13 | 14 | 39 | **1** |  |  |  |  |
| Low adherence (9-63%) | 22 | 22 | 23 | 38 | 0.69 | 0.41-1.18 | 0.69 | 0.40-1.18 |  |
| Moderate adherence >63% | 32 | 15 | 19 | 44 | 0.65 | 0.38-1.12 | 0.66 | 0.37-1.18 |  |
| High adherence >82% | 81 | 94 | 80 | 190 | 0.84 | 0.54-1.31 | 0.84 | 0.53-1.33 |  |
| All adherence | 135 | 131 | 122 | 272 | 0.78 | 0.51-1.20 | 0.78 | 0.50-1.23 |  |
| *Age, Sex, Year of treatment start, SES, Parental country of birth, Parental rhinitis (cluster robost sandwich estimator) | | | | | | | | | |

EUFOREA= European Forum for Research and Education in Allergy and Airway Diseases treatment algorithm, OR=Hazard Ratio, CI=Confidence Interval, SES=Socioeconomic status

1. **Table E5c: Any SLIT (grass or birch)**

| SENSITIVITY ANALYSIS: EUFOREA nr 3 year before treatment initiation | | | | | | |  |  |  |  |
| --- | --- | --- | --- | --- | --- | --- | --- | --- | --- | --- |
| **Any SLIT (grass or birch)** |  |  |  |  | **ALLERGIC RHINITIS SEVERITY** | | | |  |  |
|  |  | **EUFOREA** | |  | Unadjusted | | | Adjusted* | |  |
| **One year time-window** | **n** | **1** | **2** | **3** | **OR** | **95% (CI)** | | **OR** | **95% (CI)** |  |
| **Ref one dispensation** | 117 | 66 | 69 | 220 | **1** |  | |  |  |  |
| Low adherence | 132 | 86 | 76 | 219 | 0.87 | 0.69-1.09 | | 0.85 | 0.66-1.09 |  |
| Moderate adherence | 170 | 137 | 118 | 252 | 0.76 | 0.61-0.95 | | **0.70** | **0.55-0.88** |  |
| High adherence | 482 | 491 | 441 | 1024 | 0.96 | 0.80-1.15 | | 0.85 | 0.70-1.04 |  |
| All adherence | 784 | 714 | 635 | 1495 | 0.91 | 0.76-1.09 | | **0.82** | **0.67-0.99** |  |
|  |  | **EUFOREA** | |  | Unadjusted | | | Adjusted* | |  |
| **Two years time-window** | **n** | **1** | **2** | **3** | **OR** | **95% (CI)** | | **OR** | **95% (CI)** |  |
| **Ref one dispensation** | 107 | 55 | 53 | 149 | **1** |  | |  |  |  |
| Low adherence | 247 | 150 | 117 | 289 | 0.84 | 0.67-1.06 | | 0.82 | 0.65-1.04 |  |
| Moderate adherence | 172 | 139 | 107 | 247 | 0.96 | 0.76-1.21 | | 0.84 | 0.66-1.07 |  |
| High adherence | 359 | 282 | 277 | 533 | 1.00 | 0.81-1.23 | | 0.84 | 0.67-1.05 |  |
| All adherence | 778 | 571 | 501 | 1069 | 0.94 | 0.77-1.16 | | 0.83 | 0.67-1.03 |  |
|  |  | **EUFOREA** | |  | Unadjusted | | | Adjusted* | |  |
| **Three years time-window** | **n** | **1** | **2** | **3** | **OR** | **95% (CI)** | | **OR** | **95% (CI)** |  |
| **Ref one dispensation** | 107 | 44 | 25 | 111 | **1** |  | |  |  |  |
| Low adherence | 312 | 139 | 120 | 270 | 0.89 | 0.70-1.14 | | 0.84 | 0.64-1.10 |  |
| Moderate adherence | 187 | 84 | 78 | 177 | 0.96 | 0.74-1.25 | | 0.77 | 0.58-1.03 |  |
| High adherence | 280 | 168 | 142 | 285 | 1.01 | 0.79-1.29 | | 0.77 | 0.58-1.01 |  |
| All adherence | 779 | 391 | 340 | 732 | 0.95 | 0.76-1.20 | | 0.80 | 0.62-1.03 |  |
| *Age, Sex, Year of treatment start, SES, Parental country of birth, Parental rhinitis (cluster robost sandwich estimator) | | | | | | | | | | |

EUFOREA= European Forum for Research and Education in Allergy and Airway Diseases treatment algorithm, OR=Hazard Ratio, CI=Confidence Interval, SES=Socioeconomic status

**Table S6. Asthma onset for different adherence groups**

1. **Table E6a: Grass**

| SENSITIVITY ANALYSIS: EUFOREA nr 3 year before treatment initiation | | | | |  |  |
| --- | --- | --- | --- | --- | --- | --- |
| **Grass** |  |  |  | **ASTHMA ONSET** | |  |
|  |  |  | Unadjusted | | Adjusted* | |
| **One year time-window** | **No Asthma** | **Asthma** | **HR** | **95% (CI)** | **HR** | **95% (CI)** |
| **Ref one dispensation** | 158 | 23 | **1** |  |  |  |
| Low adherence | 157 | 14 | 0.65 | 0.35-1.21 | 0.60 | 0.32-1.13 |
| Moderate adherence | 229 | 35 | 1.06 | 0.65-1.75 | 0.87 | 0.51-1.46 |
| High adherence | 876 | 127 | 0.99 | 0.65-1.51 | 0.79 | 0.51-1.23 |
| All adherence | 1262 | 176 | 0.96 | 0.64-1.45 | 0.78 | 0.51-1.19 |
|  |  |  | Unadjusted | | Adjusted* | |
| **Two years time-window** | **No Asthma** | **Asthma** | **HR** | **95% (CI)** | **HR** | **95% (CI)** |
| **Ref one dispensation** | 142 | 11 | **1** |  |  |  |
| Low adherence | 267 | 32 | 1.52 | 0.80-2.92 | 1.21 | 0.60-2.42 |
| Moderate adherence | 262 | 22 | 1.17 | 0.59-2.31 | 0.79 | 0.38-1.65 |
| High adherence | 587 | 54 | 1.14 | 0.61-2.12 | 0.81 | 0.42-1.59 |
| All adherence | 1116 | 108 | 1.24 | 0.68-2.24 | 0.91 | 0.48-1.74 |

*Age, Sex, Year of treatment start, SES, Parental country of birth, Parental asthma and rhinitis and relatedness (cluster robost sandwich estimator) HR=Hazard Ratio, CI=Confidence Interval, SES=Socioeconomic status

1. **Table E6b: Any SLIT (grass or birch)**

| SENSITIVITY ANALYSIS: EUFOREA nr 3 year before treatment initiation | | | | | | |
| --- | --- | --- | --- | --- | --- | --- |
| **Any SLIT (grass or birch)** |  |  |  | **ASTHMA ONSET** | |  |
|  |  |  | Unadjusted |  | Adjusted* |  |
| **One year time-window** | **No Asthma** | **Asthma** | **HR** | **95% (CI)** | **HR** | **95% (CI)** |
| **Ref one dispensation** | 158 | 23 | **1** |  |  |  |
| Low adherence | 165 | 14 | 0.62 | 0.33-1.15 | 0.56 | 0.30-1.06 |
| Moderate adherence | 240 | 37 | 1.05 | 0.64-1.72 | 0.88 | 0.52-1.47 |
| High adherence | 931 | 128 | 0.97 | 0.64-1.50 | 0.78 | 0.50-1.22 |
| All adherence | 1336 | 179 | 0.94 | 0.62-1.41 | 0.77 | 0.50-1.19 |
|  |  |  | Unadjusted |  | Adjusted* |  |
| **Two years time-window** | **No Asthma** | **Asthma** | **HR** | **95% (CI)** | **HR** | **95% (CI)** |
| **Ref one dispensation** | 136 | 11 | **1** |  |  |  |
| Low adherence | 279 | 32 | 1.43 | 0.75-2.75 | 1.16 | 0.58-2.33 |
| Moderate adherence | 266 | 21 | 1.07 | 0.54-2.14 | 0.73 | 0.35-1.53 |
| High adherence | 605 | 54 | 1.08 | 0.60-2.01 | 0.76 | 0.39-1.50 |
| All adherence | 1150 | 107 | 1.16 | 0.64-2.11 | 0.86 | 0.45-1.65 |

*Age, Sex, Year of treatment start, SES, Parental country of birth, Parental asthma and rhinitis and relatedness (cluster robost sandwich estimator) HR=Hazard Ratio, CI=Confidence Interval, SES=Socioeconomic status

Only one treatment with SLIT

**Table S7. Allergic rhinitis severity for different adherence groups**

1. **Table E7a: Grass**

| SENSITIVITY ANALYSIS: Only those that have one treatment with SLIT | | | | | |  |  |  |
| --- | --- | --- | --- | --- | --- | --- | --- | --- |
| **Grass** |  |  |  |  |  | **ALLERGIC RHINITIS SEVERITY** | |  |
|  |  | **EUFOREA** | |  | Unadjusted | | Adjusted* |  |
| **One year time-window** | **n** | **1** | **2** | **3** | **OR** | **95% (CI)** | **OR** | **95% (CI)** |
| **Ref one dispensation** | 262 | 157 | 112 | 268 | **1** |  |  |  |
| Low adherence | 247 | 168 | 121 | 256 | 1.01 | 0.84-1.21 | 0.96 | 0.79-1.17 |
| Moderate adherence | 372 | 261 | 192 | 297 | **0.85** | **0.72-0.99** | **0.76** | **0.64-0.91** |
| High adherence | 1071 | 883 | 720 | 1087 | 1.01 | 0.86-1.16 | 0.89 | 0.77-1.04 |
| All adherence | 1690 | 1312 | 1033 | 1640 | 0.97 | 0.85-1.12 | 0.87 | 0.75-1.02 |
|  |  | **EUFOREA** | |  | Unadjusted | | Adjusted* |  |
| **Two years time-window** | **n** | **1** | **2** | **3** | **OR** | **95% (CI)** | **OR** | **95% (CI)** |
| **Ref one dispensation** | 255 | 123 | 98 | 211 | **1** |  |  |  |
| Low adherence | 566 | 294 | 198 | 388 | 0.86 | 0.73-1.01 | **0.82** | **0.69-0.98** |
| Moderate adherence | 406 | 266 | 204 | 292 | 0.93 | 0.78-1.11 | **0.81** | **0.68-0.98** |
| High adherence | 828 | 570 | 463 | 626 | 0.98 | 0.84-1.14 | **0.81** | **0.68-0.95** |
| All adherence | 1800 | 1130 | 865 | 1306 | 0.93 | 0.80-1.08 | **0.81** | **0.69-0.95** |
|  |  | **EUFOREA** | |  | Unadjusted | | Adjusted* |  |
| **Three years time-window** | **n** | **1** | **2** | **3** | **OR** | **95% (CI)** | **OR** | **95% (CI)** |
| **Ref one dispensation** | 263 | 113 | 88 | 139 | **1** |  |  |  |
| Low adherence | 773 | 317 | 239 | 364 | 0.92 | 0.77-1.09 | 0.87 | 0.72-1.04 |
| Moderate adherence | 429 | 186 | 162 | 223 | 1.02 | 0.84-1.22 | **0.82** | **0.68-0.99** |
| High adherence | 743 | 359 | 293 | 348 | 0.97 | 0.82-1.15 | **0.77** | **0.65-0.93** |
| All adherence | 1945 | 862 | 694 | 935 | 0.96 | 0.82-1.12 | **0.82** | **0.70-0.97** |
| *Age, Sex, Year of treatment start, SES, Parental country of birth, Parental rhinitis (cluster robost sandwich estimator) | | | | | | | | |

EUFOREA= European Forum for Research and Education in Allergy and Airway Diseases treatment algorithm, OR=Hazard Ratio, CI=Confidence Interval, SES=Socioeconomic status

1. **Table E7b: Birch**

| SENSITIVITY ANALYSIS: Only those that have one treatment with SLIT | | | | | | | | |  |
| --- | --- | --- | --- | --- | --- | --- | --- | --- | --- |
| **Birch** |  |  |  |  |  | **ALLERGIC RHINITIS SEVERITY** | | |  |
|  |  | **EUFOREA** | |  | Unadjusted | | Adjusted* |  |  |
| **One year time-window** | **n** | **1** | **2** | **3** | **OR** | **95% (CI)** | **OR** | **95% (CI)** |  |
| **Ref one dispensation** | 27 | 21 | 26 | 37 | **1** |  |  |  |  |
| Low adherence | 27 | 19 | 15 | 33 | 0.89 | 0.54-1.47 | 0.94 | 0.55-1.61 |  |
| Moderate adherence | 30 | 18 | 12 | 23 | 0.61 | 0.36-1.03 | 0.62 | 0.36-1.05 |  |
| High adherence | 74 | 85 | 62 | 113 | 0.98 | 0.67-1.44 | 0.99 | 0.67-1.46 |  |
| All adherence | 131 | 122 | 89 | 169 | 0.90 | 0.62-1.30 | 0.91 | 0.62-1.32 |  |
| *Age, Sex, Year of treatment start, SES, Parental country of birth, Parental rhinitis (cluster robost sandwich estimator) | | | | | | | | | |

EUFOREA= European Forum for Research and Education in Allergy and Airway Diseases treatment algorithm, OR=Hazard Ratio, CI=Confidence Interval, SES=Socioeconomic status

1. **Table E7c: Any SLIT (grass or birch)**

| SENSITIVITY ANALYSIS: Only those that have one treatment with SLIT | | | | | | | |  |  |
| --- | --- | --- | --- | --- | --- | --- | --- | --- | --- |
| **Any SLIT (grass or birch)** |  |  |  |  |  | **ALLERGIC RHINITIS SEVERITY** | | |  |
|  |  | **EUFOREA** | |  | Unadjusted | | Adjusted* | |  |
| **One year time-window** | **n** | **1** | **2** | **3** | **OR** | **95% (CI)** | **OR** | **95% (CI)** |  |
| **Ref one dispensation** | 283 | 173 | 136 | 302 | **1** |  |  |  |  |
| Low adherence | 275 | 185 | 137 | 282 | 0.96 | 0.81-1.14 | 0.93 | 0.77-1.12 |  |
| Moderate adherence | 399 | 280 | 203 | 319 | **0.81** | **0.69-0.95** | **0.75** | **0.63-0.88** |  |
| High adherence | 1142 | 960 | 780 | 1196 | 0.98 | 0.86-1.12 | 0.89 | 0.77-1.03 |  |
| All adherence | 1816 | 1425 | 1120 | 1797 | 0.94 | 0.83-1.07 | **0.87** | **0.75-0.99** |  |
|  |  | **EUFOREA** | |  | Unadjusted | | Adjusted* | |  |
| **Two years time-window** | **n** | **1** | **2** | **3** | **OR** | **95% (CI)** | **OR** | **95% (CI)** |  |
| **Ref one dispensation** | 267 | 130 | 99 | 223 | **1** |  |  |  |  |
| Low adherence | 593 | 300 | 204 | 410 | 0.86 | 0.73-1.01 | **0.83** | **0.70-0.99** |  |
| Moderate adherence | 417 | 277 | 207 | 305 | 0.94 | 0.79-1.11 | **0.83** | **0.70-0.99** |  |
| High adherence | 861 | 602 | 484 | 644 | 0.97 | 0.83-1.13 | **0.81** | **0.69-0.96** |  |
| All adherence | 1871 | 1179 | 895 | 1359 | 0.93 | 0.81-1.07 | **0.82** | **0.71-0.96** |  |
|  |  | **EUFOREA** | |  | Unadjusted | | Adjusted* | |  |
| **Three years time-window** | **n** | **1** | **2** | **3** | **OR** | **95% (CI)** | **OR** | **95% (CI)** |  |
| **Ref one dispensation** | 257 | 116 | 75 | 149 | **1** |  |  |  |  |
| Low adherence | 774 | 318 | 220 | 389 | 0.91 | 0.76-1.08 | 0.86 | 0.72-1.02 |  |
| Moderate adherence | 439 | 188 | 146 | 241 | 0.99 | 0.82-1.19 | **0.80** | **0.66-0.98** |  |
| High adherence | 743 | 360 | 260 | 384 | 0.97 | 0.82-1.15 | **0.77** | **0.65-0.92** |  |
| All adherence | 1956 | 866 | 626 | 1014 | 0.95 | 0.81-1.11 | **0.81** | **0.69-0.96** |  |
| *Age, Sex, Year of treatment start, SES, Parental country of birth, Parental rhinitis (cluster robost sandwich estimator) | | | | | | | | | |

EUFOREA= European Forum for Research and Education in Allergy and Airway Diseases treatment algorithm, OR=Hazard Ratio, CI=Confidence Interval, SES=Socioeconomic status

**Table S8. Asthma onset for different adherence groups**

1. **Table E8a: Grass**

| SENSITIVITY ANALYSIS: Only those that have one treatment with SLIT | | | | | | | | |  | |  | |  |
| --- | --- | --- | --- | --- | --- | --- | --- | --- | --- | --- | --- | --- | --- |
| **Grass** |  | |  | |  | | **ASTHMA ONSET** | | | |  | |  |
|  |  | |  | | Unadjusted | | | | Adjusted* | | | |  |
| **One year time-window** | **No Asthma** | | **Asthma** | | **OR** | | **95% (CI)** | | **OR** | | **95% (CI)** | |  |
| **Ref one dispensation** | 348 | | 52 | | **1** | |  | |  | |  | |  |
| Low adherence | 307 | | 32 | | 0.78 | | 0.52-1.18 | | 0.77 | | 0.51-1.17 | |  |
| Moderate adherence | 465 | | 68 | | 0.97 | | 0.68-1.37 | | 0.86 | | 0.60-1.23 | |  |
| High adherence | 1701 | | 243 | | 0.96 | | 0.72-1.28 | | 0.84 | | 0.62-1.13 | |  |
| All adherence | 2473 | | 343 | | 0.94 | | 0.71-1.24 | | 0.83 | | 0.62-1.12 | |  |
|  |  | |  | | Unadjusted | | | | Adjusted* | | | |  |
| **Two years time-window** | **No Asthma** | | **Asthma** | | **OR** | | **95% (CI)** | | **OR** | | **95% (CI)** | |  |
| **Ref one dispensation** | 317 | | 32 | | **1** | |  | |  | |  | |  |
| Low adherence | 599 | | 54 | | 0.95 | | 0.62-1.46 | | 0.86 | | 0.56-1.34 | |  |
| Moderate adherence | 516 | | 49 | | 1.09 | | 0.71-1.67 | | 0.90 | | 0.57-1.42 | |  |
| High adherence | 1226 | | 104 | | 0.89 | | 0.60-1.31 | | 0.69 | | 0.46-1.04 | |  |
| All adherence | 2341 | | 207 | | 0.95 | | 0.66-1.36 | | 0.78 | | 0.53-1.15 | |  |
|  |  | |  | | Unadjusted | | | | Adjusted* | | | |  |
| **Three years time-window** | **No Asthma** | | **Asthma** | | **OR** | | **95% (CI)** | | **OR** | | **95% (CI)** | |  |
| **Ref one dispensation** | 290 | | 21 | | **1** | |  | |  | |  | |  |
| Low adherence | 717 | | 52 | | 1.04 | | 0.63-1.71 | | 0.91 | | 0.55-1.53 | |  |
| Moderate adherence | 466 | | 37 | | 1.16 | | 0.68-1.96 | | 0.91 | | 0.53-1.59 | |  |
| High adherence | 882 | | 61 | | 0.98 | | 0.63-1.71 | | 0.74 | | 0.44-1.23 | |  |
| All adherence | 2065 | | 150 | | 1.04 | | 0.66-1.64 | | 0.84 | | 0.52-1.35 | |  |
| *Age, Sex, Year of treatment start, SES, Parental country of birth, Parental asthma and rhinitis and relatedness (cluster robost sandwich estimator) ) HR=Hazard Ratio, CI=Confidence Interval, SES=Socioeconomic status   1. **Table E8b: Any SLIT (grass or birch)** | | | | | | | | | | | | |  |
| SENSITIVITY ANALYSIS: Only those that have one treatment with SLIT | | | | | | | | | | | | | |
| **Any SLIT (grass or birch)** | |  | |  | |  | | **ASTHMA ONSET** | | | |  | |
|  | |  | |  | | Unadjusted | | | | Adjusted* | |  | |
| **One year time-window** | | **No Asthma** | | **Asthma** | | **OR** | | **95% (CI)** | | **OR** | | **95% (CI)** | |
| **Ref one dispensation** | | 364 | | 54 | | **1** | |  | |  | |  | |
| Low adherence | | 328 | | 33 | | 0.76 | | 0.51-1.14 | | 0.75 | | 0.50-1.13 | |
| Moderate adherence | | 484 | | 68 | | 0.92 | | 0.65-1.30 | | 0.84 | | 0.59-1.20 | |
| High adherence | | 1783 | | 249 | | 0.94 | | 0.71-1.25 | | 0.84 | | 0.62-1.13 | |
| All adherence | | 328 | | 350 | | 0.92 | | 0.70-1.21 | | 0.83 | | 0.62-1.10 | |
|  | |  | |  | | Unadjusted | | | | Adjusted* | |  | |
| **Two years time-window** | | **No Asthma** | | **Asthma** | | **OR** | | **95% (CI)** | | **OR** | | **95% (CI)** | |
| **Ref one dispensation** | | 317 | | 33 | | **1** | |  | |  | |  | |
| Low adherence | | 611 | | 53 | | 0.89 | | 0.59-1.36 | | 0.82 | | 0.53-1.27 | |
| Moderate adherence | | 527 | | 48 | | 1.01 | | 0.66-1.56 | | 0.85 | | 0.54-1.34 | |
| High adherence | | 1258 | | 103 | | 0.83 | | 0.56-1.22 | | **0.66** | | **0.44-0.99** | |
| All adherence | | 2396 | | 204 | | 0.88 | | 0.62-1.27 | | 0.74 | | 0.51-1.09 | |
|  | |  | |  | | Unadjusted | | | | Adjusted* | |  | |
| **Three years time-window** | | **No Asthma** | | **Asthma** | | **OR** | | **95% (CI)** | | **OR** | | **95% (CI)** | |
| **Ref one dispensation** | | 284 | | 21 | | **1** | |  | |  | |  | |
| Low adherence | | 715 | | 50 | | 0.98 | | 0.59-1.62 | | 0.88 | | 0.52-1.47 | |
| Moderate adherence | | 471 | | 36 | | 1.09 | | 0.64-1.86 | | 0.88 | | 0.50-1.53 | |
| High adherence | | 883 | | 61 | | 0.95 | | 0.58-1.55 | | 0.73 | | 0.44-1.21 | |
| All adherence | | 2069 | | 147 | | 0.99 | | 0.63-1.56 | | 0.81 | | 0.51-1.31 | |

*Age, Sex, Year of treatment start, SES, Parental country of birth, Parental asthma and rhinitis and relatedness (cluster robost sandwich estimator) HR=Hazard Ratio, CI=Confidence Interval, SES=Socioeconomic status
